# A core outcome set for clinical trials on non-specific low back pain: study protocol for the development of a core domain set

**DOI:** 10.1186/1745-6215-15-511

**Published:** 2014-12-26

**Authors:** Alessandro Chiarotto, Caroline B Terwee, Richard A Deyo, Maarten Boers, Chung-Wei Christine Lin, Rachelle Buchbinder, Terry P Corbin, Leonardo OP Costa, Nadine E Foster, Margreth Grotle, Bart W Koes, Francisco M Kovacs, Chris G Maher, Adam M Pearson, Wilco C Peul, Mark L Schoene, Dennis C Turk, Maurits W van Tulder, Raymond W Ostelo

**Affiliations:** Department of Health Sciences, Faculty of Earth & Life Sciences, EMGO+ Institute for Health and Care Research, VU University, de Boelelaan 1085, 1081HV Amsterdam, The Netherlands; Department of Epidemiology & Biostatistics, EMGO+ Institute for Health and Care Research, VU University Medical Center, van der Boechorstraat 7, 1081 BT Amsterdam, The Netherlands; Departments of Family Medicine, Internal Medicine, Public Health and Preventive Medicine, Oregon Institute of Occupational Health Sciences, Oregon Health and Science University, 3181 SW Sam Jackson Park Road, Portland, OR 97239 USA; The George Institute for Global Health, Sydney Medical School, The University of Sydney, PO Box M201, Missenden Rd, Kragujevac, NSW 2050 Australia; Monash Department of Clinical Epidemiology, Cabrini Institute and Department of Epidemiology and Preventive Medicine, School of Public Health and Preventive Medicine, Monash University, Melbourne, Australia - Cabrini Medical Centre, 183 Wattletree Rd, Melbourne, VIC Australia; Consumer Representative, Cochrane Collaboration Back Review Group, Maple Grove, MN 55311 USA; Masters and Doctoral Programs in Physical Therapy, Universidade Cidade de Sao Paulo, Rua Cesário Galeno 448, Sao Paulo, Brazil; Arthritis Research UK Primary Care Centre, Institute of Primary Care and Health Sciences, Keele University, Keele, Staffordshire, ST5 5BG UK; Oslo and Akershus University College of Applied Sciences, Faculty of Health Sciences and FORMI, Oslo University Hospital, Ullevaal, N-0407 Oslo, Norway; Department of General Practice, Erasmus MC, University Medical Center, PO Box 2040, 3000CA Rotterdam, The Netherlands; Spanish Back Pain Research Network (REIDE), Fundacion Kovacs, Paseo de Mallorca 36, 07012 Palma de Mallorca, Spain; Department of Orthopaedic Surgery, Dartmouth-Hitchcock Medical Center, One Medical Center Drive, Lebanon, NH03756 USA; Department of Neurosurgery, Leiden University Medical Center, Postzone J11-R, Postbus 9600, 2300RC Leiden, The Netherlands; Consumer Representative, Cochrane Collaboration Back Review Group, Newbury, MA 01951 USA; Department of Anesthesiology & Pain Medicine and Center for Pain Research on Impact, Measurement & Effectiveness, University of Washington, 1949 NE Pacific Street, Seattle, WA 98195-6540 USA

**Keywords:** Non-specific low back pain, Core outcome set, Domains, Clinical trials, Efficacy, Effectiveness, Health interventions

## Abstract

**Background:**

Low back pain (LBP) is one of the most disabling and costly disorders affecting modern society, and approximately 90% of patients are labelled as having non-specific LBP (NSLBP). Several interventions for patients with NSLBP have been assessed in clinical trials, but heterogeneous reporting of outcomes in these trials has hindered comparison of results and performance of meta-analyses. Moreover, there is a risk of selective outcome reporting bias. To address these issues, the development of a core outcome set (COS) that should be measured in all clinical trials for a specific health condition has been recommended. A standardized set of outcomes for LBP was proposed in 1998, however, with evolution in COS development methodology, new instruments, interventions, and understanding of measurement properties, it is appropriate to update that proposal. This protocol describes the methods used in the initial step in developing a COS for NSLBP, namely, establishing a core domain set that should be measured in all clinical trials.

**Methods/Design:**

An International Steering Committee including researchers, clinicians, and patient representatives from four continents was formed to guide the development of this COS. The approach of initiatives like Core Outcome Measures in Effectiveness Trials (COMET) and Outcome Measures in Rheumatology (OMERACT) was followed. Participants were invited to participate in a Delphi study aimed at generating a consensus-based core domain set for NSLBP. A list of potential core domains was drafted and presented to the Delphi participants who were asked to judge which domains were core. Participant suggestions about overlap, aggregation, or addition of potential core domains were addressed during the study. The patients’ responses were isolated to assess whether there was substantial disagreement with the rest of the Delphi panel. *A priori* thresholds for consensus were established before each Delphi round. All participants’ responses were analysed from a quantitative and qualitative perspective to ascertain that no substantial discrepancies between the two approaches emerged.

**Discussion:**

We present the initial step in developing a COS for NSLBP. The next step will be to determine which measurement instruments adequately cover the domains.

## Background

Low back pain (LBP) is the leading global contributor to years lived with disability (YLDs) and the sixth global contributor to disability-adjusted life years (DALYs) [1, 2]. The costs associated with LBP are high and they represent a substantial burden to society [3]. Approximately 90% of patients with LBP are labelled as having non-specific LBP (NSLBP), a diagnosis based on exclusion of a specific cause or pathology [4]. A wide range of health interventions for patients with NSLBP has been evaluated in clinical trials [5]. The results of clinical trials addressing similar interventions are often summarized in systematic reviews [6], but authors of these reviews have reported that outcomes are inconsistently measured and reported across trials [7, 8]. This limits ability to compare findings between studies or pool data for formal meta-analyses. Furthermore, inconsistency can be the result of selective outcome reporting bias (selective reporting of favourable outcomes) which can strongly affect the conclusions of systematic reviews [9].

To reduce heterogeneity in outcomes measured across clinical trials, development of core outcome sets (COSs) in specific health conditions has been advocated [10]. A COS is an agreed minimum set of outcomes that should be measured and reported in all clinical trials for a particular health condition [11]. A COS might increase reporting of important outcomes, reduce the risk of selective outcome reporting, and increase the feasibility of conducting meta-analyses [11]. The existence of a COS also ensures that authors of clinical trials report on outcomes that are relevant for all stakeholders [11]. Editors of Cochrane Review Groups have agreed that the availability of COSs would enhance reliability of the reviews [12].

In 1998, following an expert panel discussion held at the second international LBP Forum (The Hague, Netherlands), a proposal for a standardized set of outcomes in LBP clinical research was published [13]. Five domains were proposed for measurement (pain symptoms, back-related function, generic wellbeing, disability social role, and satisfaction with care) and specific measurement instruments were recommended for each domain [13]. This proposal has been highly cited and widely adopted over the years. However, a workshop discussion among LBP researchers during the 2012 twelfth LBP Forum (Odense, Denmark) underlined the wish to update the existing recommendations considering recent advancements in the fields of COS development and clinimetrics [11, 14]. There were two primary reasons: to explore if relevant domains were missing, and to critically appraise the recommended measurement instruments in light of a large body of newer data on measurement properties of instruments for LBP [15].

The aim of this study is to update the recommended domains for LBP clinical research [13] through the development of a COS. The Core Outcome Measures in Effectiveness Trials (COMET) [11] and the Outcome Measures in Rheumatology (OMERACT) [16] initiatives provide methodological guidance, involving a stepwise approach, to development of a COS. The first step is to determine which domains should be measured in all clinical trials (‘what’ to measure, the ‘core domain set’), and the second is to determine the instruments that should be used to assess the domains (‘how’ to measure, the ‘core outcome measurement set’) [11, 17]. This protocol presents the methods that will be adopted to reach a consensus on the core domain set.

Our goals are to present a detailed context for reporting in the results manuscript, and to provide a resource for COS developers of any health condition. Despite encouragement of the COMET initiative to make COS protocols publicly available, few have been published [18, 19], providing the motivation for this paper.

## Methods/Design

An International Steering Committee was formed to initiate and support the development of this COS. Members of the Steering Committee were chosen to represent various disciplines, geographical areas, and types of expertise. As a result, the Steering Committee includes 18 members among whom: 14 are researchers from different disciplines with ample experience in LBP clinical research (RD, CL, RB, LC, NF, MG, BK, FK, CM, AP, WP, DT, MvT, RO), three are experts in consensus-based research procedures (CT, MB, DT), six are authors of several publications in the field of clinimetrics (CT, RB, LC, FK, MG, RO), six are healthcare providers involved in the management of patients with LBP (MB, RB, LC, FK, AP, WP), and two are patient representatives (TC, MS). The members represent Europe, Australia, and North and South America.

The project team consisted of one investigator (AC), appointed to coordinate the day-to-day management of the project, and three members of the Steering Committee working at the same institution (CT, MB, RO). During face-to-face meetings, the project team established the methodology and addressed key aspects. The other members of the Steering Committee were contacted by email regarding critical decisions; when more than half of the members agreed on a decision, this was followed unless substantial and convincing arguments were raised by one or more members in disagreement. Definitions of key concepts and terms used in this study protocol follow those recently outlined by the OMERACT initiative [17] and are presented in Table 1.

**Table 1:** **Definitions of the terms used in this study protocol (Adapted from Boers** ***et al*** **.** [17]**)**

Concept	Definition
Health Condition	A situation of impaired health.
Health Intervention	An activity performed by, for, with, or on behalf of a client(s) whose purpose is to improve individual or population health, to alter or diagnose the course of a health condition, or to improve functioning.
Core Area	An aspect of health or a health condition that needs to be measured to appropriately assess the effects of a health intervention (core areas are broad concepts consisting of a number of more specific concepts called domains).
Domain or Subdomain	Component of core area: a concept to be measured, a further specification of an aspect of health, categorized within a core area.
Outcome	Any identified result in a (sub)domain arising from exposure to a casual factor or a health intervention.
Measurement Instrument	A tool to measure a quality or quantity of a variable, in this context a (sub)domain or a contextual factor.
Outcome Measurement Instrument	A measurement instrument chosen to assess outcome(s).
Core Domain Set	For study of health interventions, the minimum set of domains and subdomains necessary to adequately cover all core areas (fully measure all relevant concepts of a specific health condition within a specified scope); it describes what to measure.
Core Outcome Measurement Set	The minimum set of outcome measurement instruments that must be administered in each intervention study of a certain health condition within a specified setting to adequately cover a corresponding core domain set; it describes how to measure.
Scope	The set of factors that describes the studies and circumstances to which the core outcome set will apply. This is determined by the study questions and includes the health condition(s), target population, interventions, and so forth.
Contextual Factor	Variable that is not an outcome of the study, but needs to be recognized (and measured) to understand the study results. This includes potential confounders and effect modifiers.

### Scope of this core outcome set

The Steering Committee recommended that this COS should apply to measuring efficacy or effectiveness of health interventions in clinical trials for patients with NSLBP, defined as ‘low back pain not attributable to a recognizable, known specific pathology (eg infection, tumour, fracture, axial spondyloarthritis)’ [20]. All interventions for NSLBP are targeted by this COS, regardless of type, setting, or mode of administration. In line with COMET definition [11], this does not imply that primary outcomes of a clinical trial should always be those of the COS or that outcome measures should be restricted to the domains of the COS. However, domains of this COS should be considered for inclusion in all clinical trials on NSLBP, besides the measurement of trial-specific domains. There is no intent that this COS should be considered as a requirement for regulatory approval of drugs or devices, or that an intervention should demonstrate benefits on all COS measures to be judged efficacious or effective.

Other goals of this COS are to provide a recommendation on reporting of adverse events (AEs) in clinical trials on NSLBP and to identify contextual factors (such as confounders and effect modifiers) that should be measured alongside core domains (Table 1). However, it is out of our scope to reach consensus on contextual factors that should be measured and reported in all clinical trials. For measurement and reporting of contextual factors, we refer to the prominent work of the National Institutes of Health (NIH) Task Force that recently published a report about minimum baseline measures for clinical studies on chronic LBP [21].

### Identification of existing knowledge

The Steering Committee identified initiatives that could potentially overlap with this study at an early stage of this project. None of these initiatives had the same scope as this COS. In this subsection, we report where partial conceptual overlap between this COS and the other initiatives could lie:

1. The Initiative on Methods, Measurement, and Pain Assessment in Clinical Trials (IMMPACT) has developed a consensus-based set of outcome domains and measurement instruments for clinical trials on chronic pain [22, 23]. IMMPACT leaves ample space for the definition of more specialized COSs for some specific subgroups of patients with pain, such as this COS which focuses only on patients with NSLBP.
2. A core set for LBP based on the International Classification of Functioning (ICF) was developed by linking the health issues associated with LBP to the categories of the ICF framework [24]. These core sets are available for use in clinical studies or to guide assessment of patients with LBP, but they do not have a specific focus on the measurement of outcomes in clinical trials.
3. The recent NIH Task Force recommended baseline research standards for clinical studies on chronic LBP (CLBP) [21]. These recommendations are proposed to describe, stratify, and compare reports on patients with chronic LBP, but they are not prescriptive for the reporting of core outcomes in clinical trials.
4. The International Consortium for Health Outcomes Measurement (ICHOM) has recently developed a standardized set of outcomes for LBP [25]. The ICHOM initiative aims at targeting ‘all providers around the world’ and all LBP disorders. This suggests a rather broad scope focused on the provision of healthcare, rather than a specific focus on core outcomes for all health interventions assessed in clinical trials for NSLBP.

### Stakeholder involvement

Different stakeholders can be involved in the development of a COS: researchers, healthcare providers, patients, managers, government agencies, and industry representatives [11]. The involvement of multiple stakeholders in the consensus process for a COS is strongly recommended by COS methodologists [11, 16, 26]. For this COS, the Steering Committee decided to focus on four groups of stakeholders:

1. Healthcare researchers: these are professionals working in all fields of clinical research relevant for NSLBP (such as orthopaedics, physiotherapy, psychology, chiropractic, anaesthesiology, rheumatology, physical medicine, and rehabilitation), methodologists, or statisticians who currently work only as researchers. All researchers involved in this project should be authors of published scientific articles on clinical research for LBP.
2. Healthcare providers: these are professionals from different disciplines who have clinical experience in the management of patients with NSLBP.
3. Professionals who work both as healthcare researchers and providers: a separate category is made for these professionals because they might bring a perspective on core outcomes that may differ from that of people who perform only one of the two jobs.
4. Patients: this group is composed of people who have or have had NSLBP, and who sought healthcare for their NSLBP. Previous research has shown that it is limiting not to include patients in the development of a COS [16]. Patients have the perspective of living with the health condition and this may substantially differ from those of researchers and providers.

The decision to focus on these groups of stakeholders was based on practical considerations related to the resources of time and money available for the project. Members of the Steering Committee represented all these stakeholder groups, and representatives of each were invited to participate in the Delphi study used to reach consensus on this core domain set.

### A conceptual framework

A comprehensive framework of health can be beneficial in developing a COS, favouring the content validity of the end product. A recent review identified five conceptual frameworks that could be relevant for COSs development [27]. However, only the five Ds (discomfort, disability, drug toxicity, dollar cost, and death) and the ICF framework have been used to develop COSs in different health areas, and they cover somewhat different areas of outcome [27]. The OMERACT initiative has developed a new framework that aims at including all key aspects of a health condition to ensure comprehensiveness of COSs [17].

The OMERACT Filter 2.0 framework was created to broaden the ICF framework and combine it with Wilson and Cleary’s model of health-related quality of life [28]. The framework is subdivided into core areas (Table 1) that encompass the complete content of what is measurable in a clinical trial, including both patient-centred and intervention-specific information. It includes three core areas that describe the ‘impact of health conditions’ (‘death’, ‘life impact’, and ‘resource use and economic impact’) and one core area that describes ‘pathophysiological manifestations’. An explanation of the four core areas of the OMERACT framework is presented in Table 2. OMERACT recommends the inclusion of at least one domain from each core area in every COS [17]. However, our Steering Committee decided that, initially, no requirements related to the core areas should be applied to the domains that may become part of this core domain set. Regarding ‘death’, it was noted that this is a mandatory reporting requirement for all clinical trials, but a rare event in NSLBP. Regarding ‘pathophysiological manifestations’, it was noted that they might be out of the scope of some clinical trials, given that NSLBP is defined by its lack of known pathophysiology. The Delphi study will be used to evaluate whether substantial and convincing arguments generate further discussion on this within the Steering Committee. The OMERACT Filter 2.0 framework was used by the Steering Committee to help the development of a list of potential core domains and for discussion during the Delphi study.

**Table 2:** **OMERACT Filter 2.0 framework which specify all aspects of a health condition that should be considered in clinical trials (Adapted from Boers** ***et al*** **.** [17]**)**

Core Area	Specification
Death^a^	This core area includes possible specifications of death, such as generic or disease-specific (all-cause versus disease-specific mortality), and intervention-specific (for example, death due to surgery).
Life Impact^a^	This core area can include domains of the ICF [35] (such as activity and participation) and domains within the concept of health-related quality of life [28] (such as functional status, general health perceptions, and overall quality of life).
Resource Use/Economical Impact^a^	This core area describes the economic impact of health conditions both on society and on the individual. In fact, the presence of a health condition and its treatment incur resource use.
Pathophysiological Manifestations^b^	This core area is to assess whether or not the effect of the intervention specifically targets the pathophysiology of the health condition. Pathophysiology can include psychosocial manifestations. Example domains are: ICF body function, reversible manifestations (including modifiable risk factors and actual manifestations of ill health), and irreversible manifestations (including unmodifiable risk factors and damage). This area can also encompass all biomarkers and surrogate outcomes.

^a^These core areas belong to the concept ‘impact of health conditions’ which includes all aspects of health or a health condition that are important to the patient and society.

^b^This core area belong to the concept ‘pathophysiological manifestations of health conditions’.

### Methods to reach consensus on a core domain set for non-specific low back pain

A Delphi technique was used in this study, as in some other COS efforts [26]. This method is usually used to gain consensus among a group of experts or informed respondents that constitute the Delphi panel [29]. The respondents take part anonymously in sequential questionnaires that constitute different rounds. After each round, the group responses are fed back to the panellists who can reconsider their views based on this report of the group views [29]. The Delphi method avoids situations in which the group is dominated by the views of a few prominent personalities.

Before running the Delphi procedure, one member of the project team (AC) selected the people who were invited to be members of the panel. The project team and the Steering Committee took responsibility for drawing a list of potential core domains that was used in the Delphi study. The pre-Delphi steps described in this subsection have been completed, while the Delphi procedure itself is nearing completion.

#### Selection of panel members

We decided that at least 80 experts were required to participate in each round of the Delphi. Based on previous experience with Delphi studies [14], we calculated a minimum response rate of 40% that led to the conclusion that at least 200 people had to be invited in the first round. A list of researchers who had extensively published on LBP was compiled using an approach aimed at minimizing selection bias. First, a search in Web of Science was performed to identify authors of at least 25 publications on LBP over the last 10 years (between 2003 and 2013). Those who were authors of at least two clinical trials or one systematic review of clinical trials on NSLBP were considered eligible. One reviewer (AC) screened titles and abstracts of publications of each author against the inclusion criteria. Only researchers with a retrievable email address were selected for invitation to the Delphi procedure. To ensure that notable researchers were not excluded from this list, convenience sampling was added to the systematic search. Members of the Steering Committee were asked to indicate the names of five researchers from different disciplines that should absolutely be included in this consensus exercise. Names recommended by the Steering Committee were added to the existing list.

Healthcare providers for the Delphi panel were recruited through convenience sampling. Each member of the Steering Committee was asked to identify a minimum of 10 clinicians from different disciplines who had clinical experience in managing patients with NSLBP.

Patients were also recruited through convenience sampling. Inclusion criteria for patients were: present or past history of NSLBP, attendance of healthcare for their back complaint, and fluent understanding of written English. Three members of the Steering Committee from different geographical locations (CL, MS, and RO) were asked to identify providers who could have direct contact to patients. Patients identified by these providers were contacted by email, provided further information about the study, and invited to participate. Those who agreed were sent an informative document giving explanations of technical terms that could be encountered during the questionnaires. Those who confirmed their interest were invited to take part in the Delphi study.

All members of the Steering Committee were also invited to take part in the Delphi study. This implies that they expressed their opinion twice regarding aspects discussed previously within the Steering Committee and then in the Delphi study (for example, regarding AEs). It was decided not to exclude their opinion from the Delphi results, where responses remained anonymous and could be seen by the whole panel. We considered that this fact could outweigh the disadvantage of having their opinion counting twice for some issues. The final list of selected panellists was known only to the project team member who compiled it (AC), and was not shared with the Steering Committee or any other panellist.

#### Generation of a list of potential core domains

To make a list of (sub)domains that are routinely used as outcomes in efficacy or effectiveness trials for NSLBP, a search was performed of five recent Cochrane systematic reviews) [8, 30–32] (Oosterhuis T *et al.* unpublished data). This first list of domains was subsequently enriched by adding (sub)domains included in the comprehensive ICF core set for LBP [24] and in a conceptual model developed to characterize the burden of LBP [33]. The comprehensive ICF core set and the conceptual model on the burden of LBP were adopted in this developmental phase because they both considered the patients’ perspective [33, 34]. Subdomains that seemed to cover the same domain were grouped together to make a first draft list of domains (version one) where domains were classified into one of the core areas included in the OMERACT framework (Table 2). To find appropriate terms and definitions for each domain, we consulted the ICF Framework [35], the Health Framework of the Patient-Reported Outcomes Measurement Information System (PROMIS) [36], the Wilson and Cleary Model [28], and IMMPACT [22, 23]. When a definition was not found in one of these frameworks, it was searched in individual published papers.

Different features of the version one list of domains were discussed among the project team. A second literature search based on points of discussion was conducted and led to the development of the version two list. Feedback for version two was invited from other members of the Steering Committee, who were asked to provide critical comments on each domain. The members were also asked to indicate if there were important missing potential core domains and to indicate if they thought that certain domains were too broad or should be aggregated. The project team adjusted version two according to this input and formulated the final list of potential core domains (version three) that was used during the Delphi study.

### Delphi procedure

A minimum of two Delphi rounds (including both closed and open-ended questions) was planned *a priori*. Considering that it is not feasible to weight responses from different stakeholder groups, all panel members were invited to participate, irrespective of the number from each stakeholder group. Panel members were invited to participate in each round of the Delphi study, unless they explicitly indicated during the study that they did not wish to receive further invitations.

The project team designed the Delphi questionnaires, sent invitations and reminders to panel members, analysed the responses, and formulated the feedback reports. Questionnaires used during the whole Delphi procedure were pilot-tested by three members of the project team (CT, MB, RO) and by one or two selected panellists. For this Delphi study, the online software SurveyMonkey (SurveyMonkey, Palo Alto, United States) was used and invitations for participation were sent by email. Each round was online for three or four weeks and reminder emails were sent approximately every seven days after the initial invitation. Recommendations of a group of COS methodologists for the use of the Delphi method were followed in this study [26].

#### Delphi round one

In the first round, participants were initially given information about the study and about the formulation of the questionnaire. They completed questions about their educational and professional background, their experience with clinical research relevant for NSLBP, and whether they were invited to participate as patients. The version three list of potential core domains, subdivided into core areas, was used to rate their importance. In the survey, the order of core areas and the order of domains within core areas were randomized. Panel members were asked to indicate if each domain was important enough to be included in this core domain set; response options were ‘Yes’, ‘No’ and ‘Unsure/I do not know’. Participants were strongly encouraged to provide arguments for their choices and to suggest modifications of definitions or wording of the domains. Participants were then asked to indicate if they considered that there was a large conceptual overlap between some domains, to suggest whether some domains had be aggregated, and to suggest potential core domains not included in the list. They were also asked to indicate what they would consider to be an ideal number of domains for this COS.

Finally, participants were asked to assess whether they agreed on a specific approach for the reporting of AEs. This approach highlighted that AEs cannot be considered as one of the potential core domains because they could affect multiple core areas and domains. Therefore, only AEs occurring outside of core domains could be listed specifically as AEs, because those occurring within a defined core domain would be summarized within the results for that domain. Panellists were asked to provide reasons for their responses.

The responses of round one were analysed and collated in the feedback report. Frequencies for the response options on the importance of domains were calculated for the whole panel. Responses to open questions were checked to evaluate if substantial arguments emerged against the overall trend of frequencies. Responses of the patients’ group were highlighted and analysed separately to assess if they differed from the other panel responses.

The project team established *a priori* that domains for which more than 60% of the responders chose the response option ‘No’ and less than 20% chose the response option ‘Yes’ would be dropped from the list of potential core domains. The project team considered the suggestions for the aggregation of certain domains and the strength of the arguments. Suggested missing core domains were added to the list for the next round. Descriptive statistics were used to summarize responses on the ideal number of domains and on the reporting of AEs.

#### Delphi round two

The feedback report was provided to the panel members along with the second round of the survey. A proposal was presented for deleting domains that did not have at least 67% of first round respondents in favour or unsure about inclusion in the core domain set. Other proposals were driven by comments of the first round and concerned exclusion, aggregation, or retention of other potential core domains. For example, if in round one several comments were made about the breadth of one domain covering other more specific domains, a proposal was made to erase this broad domain. Panellists were asked to indicate whether or not they agreed with all the proposals and *a priori* consensus was set at 67% respondent agreement. Panellists were also asked to judge if missing core domains suggested in the first round were important enough to be included in the list. Response options were the same as the first round and these domains were added to the list of potential core domains if 67% of respondents were in favour or unsure of their inclusion.

Upon completion of the second round, responses were analysed and combined in the feedback report. As in the first round, patients’ responses were analysed separately to assess if they had different opinions from the rest of the panel. Results were discussed by the project team, which assessed whether substantial arguments emerged against the overall consensus obtained with quantitative answers.

#### Delphi round three

The feedback report of the second round was presented to all participants invited to the third round. Based on previous results, the potential core domains left in the list were presented in the third round to ask if each domain was indeed core. Response options were the same as the first round and participants were given the opportunity to provide arguments for their choices.

Frequencies for the response options were calculated for the whole panel and for each of the four stakeholder groups separately to evaluate if discrepancies existed between the groups. Reasons for all choices were checked to ensure that no convincing arguments were raised against the dominating group response. *A priori* consensus was set at 67% of the panel agreeing that a domain was core, with domains reaching this threshold to be included in this COS. If there were clear discrepancies between stakeholder groups or controversial arguments emerged, the results were presented to the Steering Committee that made final decisions.

### Ethical approval

As this project does not involve experiments with patients or study subjects, according to the Dutch Medical Research in Human Subjects Act (WMO), it is exempt from ethical approval in The Netherlands. No ethical approval is required in other countries from which other patients were invited to take part in the Delphi study (United States and Italy). All patients involved were asked for their consent before participation in the Delphi study, and all procedures were conducted according to the Declaration of Helsinki.

## Discussion

Development of a COS is an iterative approach that includes, firstly, determination of the domains that should be measured and reported and, secondly, determination of which outcome measurement instruments should be used to measure the domains [11, 17]. This study protocol presents the methodology that has been adopted for development of a consensus-based core domain set for clinical trials of health interventions for NSLBP. It forms the first step in updating the proposal of standardized measurement in LBP made in 1998 by Deyo *et al*. [13]. Conceptualisation and design of the second step for this COS will now need to be established, and recently published methodological guidance [37, 38] in this field could act as a useful reference for formulating the core outcome measurement set for NSLBP.

## Trial status

The third round of the Delphi study has recently been completed and the results will be analysed and reviewed by the Steering Committee. A publication reporting the results of the Delphi study will be submitted for publication in late 2014.

## Abbreviations

AE: Adverse event
COMET: Core Outcome Measures Effectiveness Trials
COS: Core outcome set
DALYs: Disability-adjusted life years
ICF: International Classification of Functioning
ICHOM: International Consortium for Health Outcomes Measurement
IMMPACT: Initiative on the Methods, Measurement, and Pain Assessment in Clinical Trials
LBP: Low back pain
NIH: National Institutes of Health
NSLBP: Non-specific low back pain
OMERACT: Outcome Measures Rheumatoid Arthritis Clinical Trials
PROMIS: Patient Reported Outcomes Measurement Information System
YLDs: Years lived with disability.
